# Recruitment methods and yield rates in a clinical trial of physical exercise for older adults with hypertension—HAEL Study: a study within a trial

**DOI:** 10.1186/s12874-022-01535-7

**Published:** 2022-02-10

**Authors:** Cíntia E. Botton, Lucas P. Santos, Bruna G. Moraes, Raíssa B. Monteiro, Maria Laura B. Gomes, Eurico N. Wilhelm, Stephanie S. Pinto, Daniel Umpierre

**Affiliations:** 1 National Institute of Science and Technology for Health Technology Assessment (IATS), Hospital de Clínicas de Porto Alegre, Porto Alegre, RS Brazil; 2 Exercise Pathophysiology Research Laboratory, Clinical Research Center, Hospital de Clínicas de Porto Alegre, Porto Alegre, RS Brazil; 3 Institute of Physical Education and Sports, Federal University of Ceará, Fortaleza, CE Brazil; 4 Graduate Program in Cardiology and Cardiovascular Sciences, Universidade Federal Do Rio Grande Do Sul, Porto Alegre, RS Brazil; 5 Neuromuscular Evaluation Laboratory, Universidade Federal de Pelotas, Pelotas, RS Brazil; 6 Department of Sport, Exercise and Rehabilitation, Northumbria University, Newcastle upon Tyne, UK; 7 Department of Public Health, Universidade Federal Do Rio Grande Do Sul, Porto Alegre, RS Brazil

**Keywords:** Physical activity, Randomized clinical trial, Lifestyle intervention, Recruitment approaches

## Abstract

**Background:**

Although the prevalence of hypertension is high in older adults, clinical trial recruitment is a challenge. Our main aim was to describe the HAEL Study recruitment methods and yield rates. The secondary objectives were to explore the reasons for exclusion and to describe the characteristics of the enrolled participants.

**Methods:**

This is a descriptive study within a trial. The HAEL Study was a Brazilian randomized two-center, parallel trial with an estimated sample of 184 participants. The recruitment strategy was based on four methods: electronic health records, word of mouth, print and electronic flyer, and press media. The yield rate was the ratio of the number of participants who underwent randomization to the total number of volunteers screened, calculated for overall, per recruitment method, by study center and by age group and sex. Additionally, we described the reasons for exclusion in the screening phase, as well as the demographic characteristics of those enrolled. The data are presented in absolute/relative frequencies and mean ± standard deviation.

**Results:**

A total of 717 individuals were screened, and 168 were randomized over 32 months. The yield rate was higher for word of mouth (30.1%) in the overall sample. However, press media contributed the most (39.9%) to the absolute number of participants randomized in the trial. The coordinating center and participating center differed in methods with the highest yield ratios and absolute numbers of randomized participants. The main reason for exclusion in the screening phase was due to the physically active status in those intending to participate in the study (61.5%). Out of 220 participants included, 52 were excluded mainly because they did not meet the eligibility criteria (26.9%). Most of the screened volunteers were women (60.2%) age 60–69 years (59.5%), and most of the randomized participants were Caucasian/white (78.0%).

**Conclusions:**

Multiple recruitment methods constituted effective strategies. We observed that approximately one of every four individuals screened was allocated to an intervention group. Even so, there were limitations in obtaining a representative sample of older Brazilian adults with hypertension. Data show an underrepresentation of race and age groups.

**Trial registration:**

This SWAT was not registered.

## Background

The prevalence of hypertension is increasing worldwide [1]. Approximately 30% of the Brazilian population has hypertension [2], and in older adults, the prevalence is twice as high [3]. Structured physical exercise, as a nonpharmacological intervention, produces cardiovascular health benefits and is considered a cornerstone for hypertension management [4–6]. Clinical trials are essential to understand the effectiveness of physical activity as part of antihypertensive treatments, but few studies have been designed that exclusively include older adults with hypertension.

Although the total number of older individuals with hypertension is substantial, a successful recruitment process for clinical trials is not guaranteed. Identifying and recruiting potential research participants is a common challenge for studies and is considered a determining factor for trial success [7]. Especially in some settings, recruiting participants can be an operational barrier to clinical trials, particularly those conducted in developing countries, as financial costs associated with complex and lengthy administrative processes are an additional barrier to trial completion [8]. In addition, the recruited sample is not always representative, and the external validity of clinical trials remains a great challenge [7, 9].

Many recruitment strategies exist to target potential research participants, and although some previous studies tried to explore these in different fields [10–13], it is unclear which strategies were the most useful. The effectiveness of recruitment strategies depends on population factors, such as physical, demographic and clinical characteristics, as well as the trial setting and type of study intervention [14]. Hence, analyses of the yield rate of recruitment methods may be highly informative for future studies [10, 11, 13], especially those methods conducted with a limited research budget.

To share challenges and outputs, our general purpose was to describe the recruitment strategies for the Hypertension Approaches in the Elderly: a Lifestyle Study (HAEL Study) conducted in southern Brazil. Our primary aim was to describe the yield rates calculated for the overall HAEL Study, per study center and recruitment method, and considering age groups and sex. Additionally, we calculated the crude recruitment output. Our secondary objectives were to explore the reasons for exclusion throughout the screening phase and for non-enrolled participants who signed the consent form. Finally, we describe the participants’ demographic characteristics who underwent randomization.

## Methods

### Study design

This is a descriptive study within a trial (SWAT). We did not register the study previously, although we preplanned to perform this analysis and therefore collected all data related to the recruitment phase. At the end of the screening call, participants verbally declared their consent to the use of their eligibility screening data. From eligible volunteers, those who agreed to participate in the study signed a consent form before starting baseline data collection. The study was approved by the Ethics Committee/IRB from the Hospital de Clínicas de Porto Alegre (CAAE: 62,427,616.0.1001.5327) and Universidade Federal de Pelotas (CAAE: 62,427,616.0.2001.5313). The study was conducted according to the World Medical Association Declaration of Helsinki.

### HAEL study (host study)

This SWAT is nested within the HAEL Study, which was a randomized, single-blinded, multicenter, two-arm, parallel, superiority trial. The study was designed to evaluate the efficacy of a combined aerobic and resistance exercise training program on reducing blood pressure levels compared with a control group undergoing health education in older patients with hypertension (≥ 60 years old). The study was prospectively registered (Clinicaltrials.gov NCT03264443), and the complete protocol was published [15]. Recruitment data were collected at both centers where the study was conducted, located in southern Brazil. The coordinator center (CC) was based in Porto Alegre, the largest city in the state of Rio Grande do Sul, at the Hospital de Clínicas de Porto Alegre. The participating center (PC) was based in Pelotas, the fourth most populous city in the Rio Grande do Sul, located 168 mi from Porto Alegre, at the Universidade Federal de Pelotas. The recruitment period was from August 2017 to March 2020. In the PC, the recruitment ended in March 2019.

### Sample size

All individuals who were screened for HAEL study eligibility by telephone were included in this study. The HAEL study sample was based on two studies [16, 17]. We estimated that 184 participants would provide power values of 0.79 and 0.92 to detect difference of 2.5 mmHg and 3.0 mmHg between the two group mean values for the 24-h systolic blood pressure, with an expected standard deviation of 6.0 mmHg. A two-sided test with a significance level of 0.050, obtained from a mixed-effects model fit without the treatment-by-center interaction, was considered. More details about the sample size calculation are in the HAEL study protocol [15].

### Eligibility criteria and reasons for exclusion

In the telephone screening and after signing the consent form, for participants not enrolled, the reasons for exclusion were considered according to the eligibility criteria or other reasons that were identified during the baseline test period. The inclusion criteria were as follows: 1) diagnosis of hypertension as assessed by previous ambulatory blood pressure monitoring (no later than six months) or current use of antihypertensive drugs; 2) age ≥ 60 years; 3) unchanged pharmacological scheme for four weeks prior to enrollment; and 4) willingness to participate in either intervention group. Exclusion criteria included 12 characteristics that could increase the cardiovascular risk during exercise (e.g., cardiac event within the 12 recent months) or modify (increasing or decreasing) intervention adherence due to external factors. Additionally, the exclusion criterion “to be physically active” was not described in the study protocol [15] but was considered in the eligibility process. Those who performed ≥ 30 min of physical activity at moderate intensity, at least three days/week, in the last three months before screening were excluded.

### Recruitment methods

The recruitment strategy was based on four preplanned approaches: 1) *electronic health records* from public health care units; 2) *word of mouth*; 3) *print and electronic flyer*; and 4) *press media*. For the *electronic health records*, the lists of patients registered in one/two basic care units of the public health system were accessed. The *word-of-mouth* method comprises word-of-mouth referrals from friends, relatives, or professionals. Professional referrals were considered when specialists (i.e., cardiologists, gerontologists, etc.) directed potential participants to the study. The *print and electronic flyer* method included disseminating flyers with standard information about the research study and contact information. Flyers were distributed in print on the streets, and flyer posters were placed in pharmacies and grocery shops. Additionally, the flyer was released in digital format on social media (i.e., Facebook and Instagram) and WhatsApp Messenger. Finally, *press media* was a method of recruitment through free advertisement in local and widely circulated newspapers. During the telephone screening, potential participants were asked how they learned of the study and the information was used to determine which recruitment strategy reached them. Although the same approaches were used in both centers, each center was free to decide which methods would be prioritized.

### Participants’ demographic characteristics assessment

Participants completed questionnaires to self-identify their sex (i.e., male or female), race/ethnicity, and age (years). From the participants’ characteristics, the categories of race/ethnicity were created as follows: Caucasian/white, black/Afro-descendants, Asian, indigenous, other/mixed.

### Data analysis

Descriptive statistical analyses were used to assess the study results. Continuous data are presented as the means and standard deviations. Categorical data are presented in absolute and relative frequencies. We calculated the yield rate by the ratio of the number of participants who underwent randomization to the total number of volunteers that were screened (i.e., yield rate = individuals randomized/individuals screened). First, we calculated the overall yield rate of the trial. Second, we calculated the yield rate per recruitment method and study center. Last, the yield rate was estimated per recruitment method, stratified by age group and sex. In addition to the yield rate, we calculated the crude recruitment output by the ratio of those who underwent randomization per method to the total number of participants randomized (i.e., crude recruitment output = individuals randomized per method/total of individuals randomized). Exclusion reasons were counted for all contacts made in the telephone screening and for those who were not enrolled in the study after signing the consent form. We had missing data because some participants refused to answer the form completely during the telephone screening or due to failure to complete it. The missing data were treated as undefined. All descriptive analyses were generated in Microsoft Excel software, 2016 (Microsoft Inc., Redmond, WA, USA), and IBM SPSS Statistics version 21.0 (IBM SPSS Inc., Chicago, IL, USA).

## Results

**Fig. 1:**
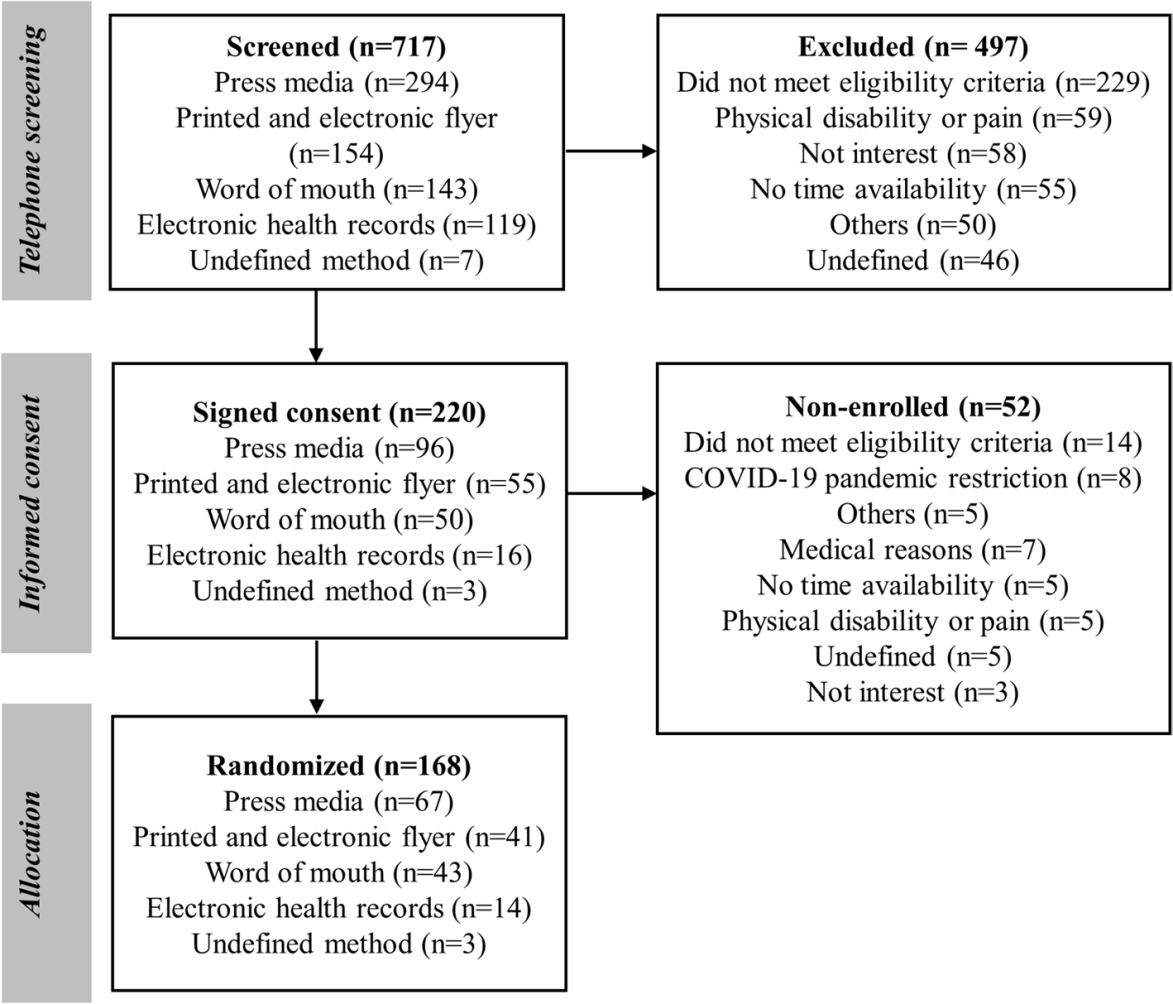
Flowchart of the recruitment process for the HAEL Study

The study flowchart per recruitment method is described in Fig. 1. Throughout the study enrollment process, 717 individuals were telephone-screened for eligibility. On average, over the 32 months of the study, 22 to 23 individuals were screened each month, and five to six were randomized. Most individuals were screened by the CC (487; 67.9%) compared to the PC (210; 29.5%), and 20 (2.7%) had an undefined center. Between telephone screening and face-to-face interviews, 69.3% (CC, *n* = 326; PC, *n* = 151; undefined center, *n* = 20) were excluded or declined to participate, and 220 (30.6%) signed the consent form. Through baseline data collection and before allocation, 52 individuals were excluded or declined to participate. In total, 168 participants were randomized: 119 (70.8%) in the CC group and 49 (29.2%) in the PC group.

For CC and PC, 244 (50.1%) and 36 (17.1%) individuals screened were reached by *press media*, respectively. *Printed and electronic flyer* reached 139 (28.5%) and 14 (6.6%) screened individuals, while *word of mouth* reached 92 (18.9%) and 50 (23.8%) individuals for CC and PC, respectively. *Electronic health records* accounted for nine individuals (1.8%) screened in the CC and 110 (52.4) in the PC.

### Demographic characteristics of the enrolled participants

For participants who underwent randomization, the overall sample age range was from 60 to 84 years old, and most of them were female (61.9%) and Caucasian/white (78%) (Table 1).

**Table 1:** Demographic characteristics of randomized participants

Characteristics	Total	CC	PC
Age, *year (mean* ± *SD)*^a^	67.3 ± 5.5	67.4 ± 5.6	67.1 ± 5.2
Sex, *n (%)*^a^			
Female	104 (61.9)	66 (55.5)	38 (77.6)
Male	64 (38.1)	53 (44.5)	11 (22.4)
Race/ethnicity, *n (%)*^b^			
Caucasian/white	131 (78)	96 (80.7)	35 (71.4)
Black/Afro-descendants	28 (16.7)	16 (13.4)	12 (24.5)
Asian	1 (0.4)	1 (0.8)	-
Indigenous	3 (1.8)	2 (1.7)	1 (2.0)
Other/mixed	3 (1.8)	2 (1.7)	1 (2.0)

^a^Based on the total sample (*n* = 168), ^b^Based on 166 individuals of total sample

### Yield rate

**Fig. 2:**
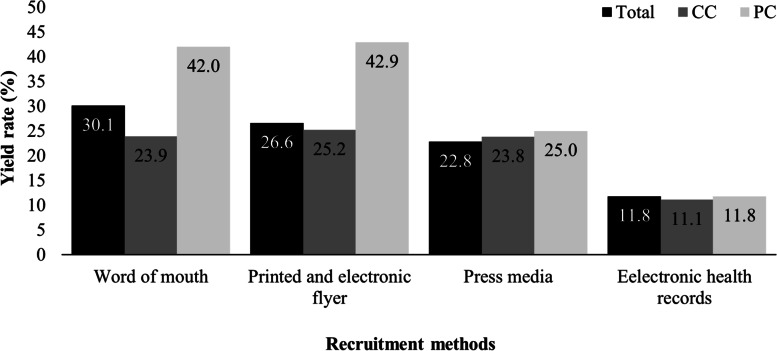
Yield rate per recruitment method (i.e., yield rate = individuals randomized/individuals screened). CC = coordinator center; PC = participating center

The overall yield rate was 23.4% (Fig. 2). Separately, the yield rate was 24.4% for the CC and 23.3% for the PC. Twenty and seven individuals had undefined centers and recruitment methods, respectively, and were excluded from the stratified yield rate analysis. The yield rate per recruitment method was higher for *word of mouth* (30.1%) for the overall study and *printed and electronic flyer* for the CC (25.2%) and the PC (42.9%). The lowest yield rate was determined by *electronic health records*.

### Yield rate per sex and age range

For 10 and 13 individuals screened, sex and age, respectively, were undefined. Most of the screened individuals were female (432; 60.2%) compared to male (275; 38.3%). Relative to age range, 427 (59.5%) individuals ranged from 60–69 years, 221 (30.2%) from 70–79 years, 40 (5.6%) from 80–97 years and 16 (2.2%) ranged from 42–59 years and were excluded because they did not meet the age requirement of the eligibility criteria (Table 2). Thirteen individuals with undefined ages were not counted.

**Table 2:** Yield rate per recruitment method, considering age groups and sex

	**Electronic health records**		**Word of mouth**		**Printed and electronic flyer**		**Press media**	
	**Total, n (%)**	**YR (%)**	**Total, n (%)**	**YR (%)**	**Total, n (%)**	**YR (%)**	**Total, n (%)**	**YR (%)**
Sex								
Female	85 (19.6)	15.3	102 (23.6)	32.3	104 (24.0)	26.0	137 (31.7)	20.4
Male	29 (10.5)	3.4	39 (14.2)	25.6	50 (18.2)	28	154 (56.0)	25.3
Age range								
60–69 years	76 (17.8)	14.5	84 (19.7)	34.5	101 (23.6)	25.7	165 (38.6)	28.5
70–79 years	29 (13.1)	10.3	46 (20.8)	26.1	44 (19.9)	34.1	99 (44.8)	16.2
80–97 years	8 (20.0)	0.0	6 (15.0)	33.3	2 (5.0)	0.0	24 (60)	16.6

*YR* Yield rate

*Press media* was the method that mostly reached males (56.0%) and females (31.7%). For females, the method with the highest yield rate *was word of mouth* (32.4%), whereas for males, it was *printed and an electronic flyer* (28.0%). The lowest yield rate strategy was *electronic health records* for both females (15.3%) and males (3.4%). For the age groups of 60–69 years and 80–97 years, the *word of mouth* method had the highest yield rate (34.5% and 33.3%, respectively). The age group ranging from 70–79 years had the highest value with *printed and electronic flyer* (34.1%).

### Crude recruitment output per method

**Fig. 3:**
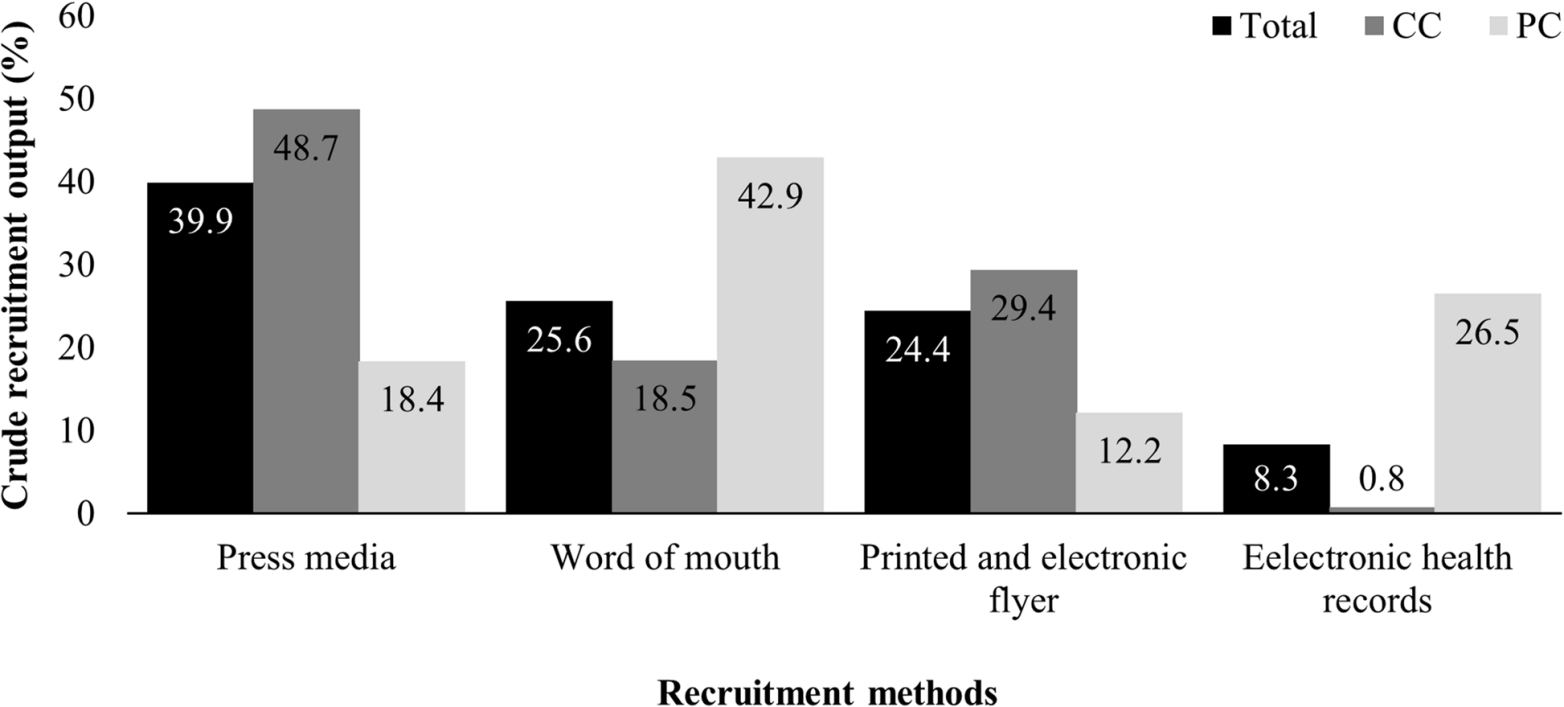
Crude recruitment output per recruitment methods (i.e., crude recruitment output = individuals randomized per method/total of individuals randomized). CC = coordinator center; PC = participating center

The method that contributed most to the total participants who underwent randomization was *press media* (39.9%) (Fig. 3). Considering centers separately, *press media* (48.7%) was also for the CC, while *word of mouth* (42.9%) was for the PC. The lowest crude output was *electronic health records* for the overall study (8.3%) and for the CC (0.8%). For the PC, *printed and electronic flyer* had the lowest crude recruitment output (12.2%).

### Reasons for exclusions

Out of 497 (69.3%) individuals excluded between telephone screening and face-to-face interviews, 229 (46.1%) did not meet the eligibility criteria. The main eligibility criteria that caused exclusion were: 1) Physical activity (141; 61.5%); 2) Age < 60 years old (15; 6.5%); 3) No diagnosis of hypertension assessed according to the study’s criteria (12; 5.2%); 4) History of myocardial infarction, revascularization procedures, deep vein thrombosis, cerebrovascular events or pulmonary embolism (12; 5.2%). Eligibility criteria were undefined for 15 individuals. Other exclusion criteria, such as cancer, heart failure, pulmonary disease, kidney disease or neurological disease, unwillingness to participate in either one or both of the intervention groups, excessive consumption of alcoholic drinks, or another person from the same household/family participating in the study, were less frequent (i.e., 1 to 6 individuals). Additionally, 58 (11.7%) individuals were not interested in the study, 59 (11.9%) had pain or physical disability, and 55 (11.1%) had no time available. Other reasons accounted for 4.4% of the exclusions (*n* = 22), and the reasons were undefined for 46 (9.2%) participants.

### Reasons for nonenrolment

After signing the consent form and at baseline data assessments, 52 (23.6%) of the 220 participants were not enrolled. Fourteen individuals (26.9%) did not meet the eligibility criteria (i.e., one had individual plans to move to another city during the period of participation; 10 had medical reports indicating moderate or high risk for exercise-related events based on the initial maximal exercise test and clinical evaluation; and three were physically active). Seven individuals (13.5%) had different medical reasons for exclusion, and eight (15.4%) were restrained from continuing the study due to the restrictions of the COVID-19 pandemic. Five individuals (9.6%) had physical disability or pain, five (9.6%) had no time available, and three were no longer interested in participating (5.8%). Other reasons accounted for 9.6% (*n* = 5) of exclusions, and five individuals (9.6%) had undefined exclusion reasons.

## Discussion

The general purpose of this study was to assess the recruitment strategy in the HAEL study. We observed that approximately one individual for every four individuals screened was allocated to an intervention group. The yield rate was higher with *word of mouth* (30.0%), but the *press media* had the highest crude recruitment output (39.8%). The highest yield rate strategies were *printed and electronic flyer*, *word by mouth,* and *press media*, in this order, for both centers separately. In contrast, *electronic health records* were the approach with the lowest yield rate.

Interestingly, *press media* was the main driver of absolute screening and recruitment at the CC, which may be (i) related to the newspapers’ reach in which recruitment was advertised and (ii) especially due to active support from the hospital's communication division at the CC, which favored contact with several newspapers. Notably, newspapers do not usually charge fees to advertise notes of study recruitment in the state where the study was located. At the PC, the *electronic health records* method contributed to the screening of several individuals, which was apparently facilitated by the existing relationships between university researchers and health services professionals, who were more readily engaged to identify potentially eligible individuals.

For the overall study sample, the method with the highest yield rate was *word of mouth*. In addition, by assessing the crude randomization output, which ultimately completes the study sample, both *press media* and *word of mouth* were important sample sources for CC and PC, respectively. Thus, consistent with previous studies [13, 18], it was essential to use varied recruitment strategies to reach the estimated number of individuals in our trial. We highlight that the *word of mouth* method resulted in a high yield rate as well as crude recruitment output at the PC. This might suggest that referrals are a relevant and more effective approach than *press media* in smaller cities.

Some recruitment strategies used in our trial, such as the use of *electronic flyers* or *press media*, were implemented with minimal initial effort. However, all methods except *electronic health records* required potential participants to initiate contact with the research team. This could have biased the sample toward individuals highly motivated to exercise or be healthy, which may partly reduce the generalizability of the results. Even using three widely disseminated recruitment strategies (*press media, word of mouth, printed or electronic flyer),* approximately 60% of the individuals screened were females and were between 60 and 69 years old (both sexes), whereas few individuals aged between 80 and 97 years were screened (*n* = 40) or randomized (*n* = 6). The chance of having health complications and limitations to participate in a clinical trial is greater as aging progresses. Furthermore, older individuals may have more barriers (e.g., regarding willingness or commuting) to participate in clinical trials [19]. Additionally, most of the randomized participants self-identified as Caucasian/white (78%), while a minority were black (16.7%). Therefore, these data show an underrepresentation of race/ethnicity and age groups that may not properly represent the target population [20, 21].

The yield rates from the recruitment methods differed between sexes, with *word of mouth* resulting in more females being randomized (approximately 1 out of 3), whereas *printed and electronic flyer* resulted in more males (approximately 1 out of 4). It is worth mentioning that even when using four different recruitment methods, *press media* accounted for 56% of screened men. In the age groups of 60–69 and 80–97 years, the *word of mouth* method had the highest yield rate, whereas *printed and electronic flyer* achieved higher rates among individuals age 70–79 years. To understand that methods vary regarding sex and age may be useful to targeted recruitment in future studies.

Even when using a 32-month recruitment period, the pretrial calculated sample size (*N* = 184) was not fully achieved, lacking inclusion/randomization of 16 individuals. Based on the CC yield rate (24.4%), approximately 65 additional individuals would need eligibility screening. Due to the COVID-19 pandemic, the trial was terminated after a careful assessment that included external advice. However, other factors also made recruitment challenging. The PC found more barriers that hindered the study and ended the recruitment process sooner than expected. Apparently, barriers were mainly related to institutional differences regarding support clinical studies, infrastructure, and human resources (team size). We reason that strategies specific to each study center and recruitment process could mitigate these barriers and reduce differences between the centers.

The number and restriction of eligibility criteria for clinical trials may limit recruitment [22]. As an attempt to recruit a representative sample, we reduced the exclusion criteria to characteristics that would represent a risk factor for exercise. The main reason for exclusion at telephone screening was individuals declaring to be physically active, comprising 20% of the individuals who sought information about the study. Other usual reasons for exclusion were the occurrence of pain, physical disability, and not having a sufficient amount of time available. Even though the training program allowed some changes, depending on the level of physical disability, individuals could not completely comply with the protocol, so this was listed as an exclusion criterion. Therefore, anticipating the main sources of exclusions may help to design recruitment notes and objectively address such criteria in eligibility screening.

Finally, approximately 25% of the individuals who signed the consent form were excluded. Some individuals showed cardiovascular conditions identified only when the stress test was performed. Other cases were individuals who omitted crucial information during the face-to-face interview, such as being physically active or leaving the study without explanation. The non-enrollment of participants is routine in any trial, but it can be disadvantageous, as there is an investment of financial and human resources. Therefore, future studies may try to refine the initial eligibility process before the start of baseline testing to avoid wasting resources.

The present study has limitations. First, we did not estimate the costs for each method, which is important considering that many groups would expect to pay advertising costs in press media, which was not the case in our trial. Second, although we used different recruiting strategies between centers, there were differences that were not exhaustively explored regarding opportunities to advertise the trial in each center. Third, although unrelated to our methods, we experienced limitations in reaching a representative sample of older Brazilian adults with hypertension. We speculate that with wider access to *electronic health records,* we would have recruited a sample of more diverse participants; however, this claim needs further assessment.

## Conclusions

In summary, using multiple methods to recruit participants helped reach older adults to participate in the HAEL Study, in which calls in *press media* and *word of mouth* were valuable approaches in both study centers. However, none of the methods showed a clear advantage in yielding randomization of older (> 70 years old) or black participants. We believe that our experience can help future studies on physical exercise and help to recruit older adults with hypertension.

## Data Availability

The datasets used and/or analyzed during the current study are available from the corresponding author on reasonable request.

## Abbreviations

CC: Coordinator center
HAEL: The Hypertension Approaches in the Elderly
PC: Participating center
